# Hyperbaric oxygen therapy for spinal cord injury: A protocol for systematic review and meta-analysis

**DOI:** 10.1097/MD.0000000000023536

**Published:** 2020-12-04

**Authors:** Tong Li, Yiran Wang, Chaoqun Feng, Qianchun Li, Qiang Ran, Botao Chen, Yang Yu, Leiming Jiang, Xiaohong Fan

**Affiliations:** Hospital of Chengdu University of Traditional Chinese Medicine, Chengdu, Sichuan Province, PR China.

**Keywords:** hyperbaric oxygen therapy, meta-analysis, protocol, spinal cord injury

## Abstract

**Background:**

Hyperbaric oxygen (HBO) therapy can prevent further spinal cord injury (SCI) caused by spinal cord ischemia-reperfusion injury to the maximum extent, which has been reported increasingly in recent years. However its security and effectiveness still lack of high-quality medical evidence. In this study, we will perform a systematic review of previously published randomized controlled trials (RCTs) to evaluate the efficacy and safety of HBO therapy for SCI.

**Methods:**

All potential RCTs on HBO therapy for SCI will be searched from the following electronic databases: PubMed, Embase, Cochrane Library, Web of Science, China National Knowledge Infrastructure, Chinese Science and Technology Periodical Database, Wanfang database and Chinese Biomedical Literature Database. We will search all electronic databases from their initiation to the September 30, 2020 in spite of language and publication date. Two contributors will independently select studies from all searched literatures, extract data from included trials, and evaluate study quality for all eligible RCTs using Cochrane risk of bias tool, respectively. Any confusion will be resolved by consulting contributor and a consensus will be reached. We will utilize RevMan 5.3 software to pool the data and to conduct the data analysis.

**Results:**

The quality of the assessments will be assessed through Grading of Recommendations Assessment, Development, and Evaluation. Data will be disseminated through publications in peer-reviewed journals.

**Conclusion:**

This study will provide evidence to evaluate the efficacy and safety of HBO therapy for SCI at evidence-based medicine level.

**Trial registration number:**

INPLASY 2020100084.

## 1 Introduction

Spinal cord injury (SCI) refers to the complete or partial loss of spinal nerve function caused by a variety of reasons. It contains a series of pathological injury links, which often lead to serious consequences, ranging from partial limb paresthesia and incontinence to paraplegia and even brain death.^[1–5]^ Hyperbaric oxygen (HBO) therapy is a safe and noninvasive physical therapy that involves placing patients in a pressurized chamber and inhaling high or pure oxygen at higher than atmospheric pressure to improve dissolved oxygen in the blood to relieve symptoms and treat disease with fewer side effects.^[6,7]^ It is mainly aimed at secondary spinal cord injury, which can prevent further spinal cord injury caused by spinal cord ischemia-reperfusion injury to the maximum extent, inhibit cell apoptosis and autophagy, and effectively promote nerve fiber regeneration.

In recent years, hyperbaric oxygen therapy for spinal cord injury has been reported increasingly,^[8–28]^ but there is still a lack of high-quality meta-analysis. Therefore, this study is expected to provide more systematic and comprehensive evidence to evaluate the efficacy and safety of HBO therapy for SCI.

## 2 Methods

### 2.1 Design and registration of the Study

All methods will conform to Preferred Reporting Items for Systematic Reviews and Meta-Analyses (PRISMA) protocols guidance^[29,30]^ and has been registered in INPLASY (2020100084). All data analysis will adhere to the PRISMA statement.^[31,32]^

### 2.2 Ethics and dissemination

All data will be disseminated through publications in peer reviewed scientific journals. The study includes no primary patient data or patient identifiers meaning local ethical approval is not required.

### 2.3 Eligibility criteria

#### 2.3.1 Types of studies

Randomized controlled trials (RCTs) will be included in this systematic review regardless of publication status and language.

#### 2.3.2 Types of participants

Any adult patients (≥18 years) diagnosed with SCI will be included in this study regardless their ethnicity, sex, age, and the length and severity of disease.

#### 2.3.3 Types of interventions

The experimental group is hyperbaric oxygen therapy combined with conventional treatments such as surgery, drugs and rehabilitation physiotherapy. The control group received conventional treatments such as surgery, drugs and rehabilitation physiotherapy.

#### 2.3.4 Type of outcome measurements

##### 2.3.4.1 Primary outcomes

1. American Spinal Injury Association (ASIA) Score: Includes American Spinal Injury Association Impairment Scale (AIS) and Asia Motor Score (AMS)
2. Frankel Classification Grading System
3. Functional Independence Measure (FIM)
4. Barthel Index

##### 2.3.4.2 Secondary outcomes

1. Hospitalization Time
2. Death Rate
3. Incidence of Any Expected or Unexpected Adverse Event
4. Hamilton Anxiety Rating Scale (HAM-A)
5. Hamilton Depression Rating Scale (HAM-D)

#### 2.3.5 Exclusion criteria

Republished literature, unable to obtain full text or data, the type of study could not be confirmed, quasi-randomized controlled trials (QRCTs), animal trials, retrospective studies and other nonrandomized studies will be excluded.

### 2.4 Search strategy

#### 2.4.1 Electronic database searches

All the major electronic databases will be searched from inceptions to the September 30, 2020. The basic search strategy is outlined in Table 1.

**Table 1:** Search Strategy For PubMed.

#1	Spinal Cord Injuries [Mesh]
#2	(Spinal Cord Injury) OR (Spinal Cord Injuries) OR (Spinal Cord Trauma) OR (Spinal Cord Traumas) OR (Spinal Cord Transection) OR (Spinal Cord Transections) or (Spinal Cord Laceration) OR (Spinal Cord Lacerations) OR (Spinal Cord Contusion) OR (Spinal Cord Contusions) OR (Traumatic Myelopathy) OR (Traumatic Myelopathies) OR (Post-Traumatic Myelopathy) OR (Post-Traumatic Myelopathies) OR (SCI)
#3	#1 OR #2
#4	Hyperbaric Oxygenation [mesh]
#5	(Hyperbaric Oxygenations) OR (Hyperbaric Oxygen) OR (Hyperbaric Oxygen Therapy) OR (Hyperbaric Oxygen Therapies) OR (HBO)
#6	#4 OR #5
#7	(surgery) OR (drug) OR (methylprednisolone) OR (rehabilitation) OR (physiotherapy) OR (therapy) OR (Physical therapy)
#8	Randomized controlled trial OR clinical study OR Clinical Trial OR Controlled study OR Controlled Trial OR Random^∗^Control^∗^ study OR random^∗^ Control^∗^ Trial
#9	#3 AND #6 AND #7 AND #8

Mesh = medical subject heading.

#### 2.4.2 Search for other resources

To avoid missing potential trials, we will also retrieve conference papers, dissertations, ongoing studies, and reference list of all related reviews.

### 2.5 Data collection and analysis

#### 2.5.1 Study selection

**Figure 1:**
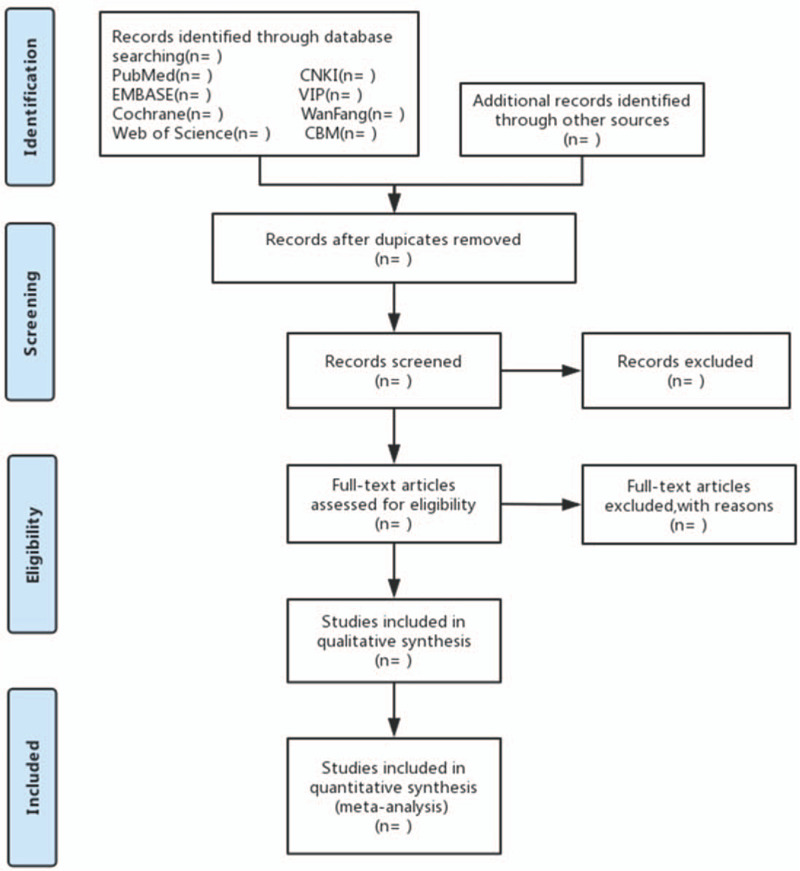
Flowchart of literature selection.

Selection of studies will be independently conducted by 2 contributors and will be cross-checked between them. Any different views between them will be solved by discussion with the help of another contributor. All collected documents will be imported into EndNote X9 and all duplicated literatures will be removed. Then, titles and abstracts of all records will be scanned to rule out obvious nonconformities. After that, we will obtain full-texts of remaining studies and will carefully examine them according to the eligibility criteria. The entire filtering procedure will be presented in a flowchart (Fig. 1).

#### 2.5.2 Data extraction and management

Data will be extracted by 2 contributors using a predetermined sheet of data collection. The extracted data includes title, first author, publication year, sample size, patient characteristics (such as race, sex, age, eligibility criteria, and severity and duration of SCI), trial setting, trial methods (such as details of randomization and blind), specifics of treatment and comparators, outcomes, safety, and other relevant data. If we identify any missing or incomplete data, we will contact original corresponding authors to request them. Data extraction procedures will be assessed by a third reviewer.

#### 2.5.3 Quality assessments

Two contributors will perform quality assessments and review the risk of bias using the Cochrane Collaboration's risk-of-bias assessment method (v6).^[33]^ This scale includes 7 risk of bias items, and each will be described as low, unclear, or high risk. Data will be presented in the risk of bias graph. Discrepancies will be resolved or as required, by a third reviewer.

#### 2.5.4 Measurement of treatment effect

To evaluate the treatment effect for continuous data, mean difference or standardized mean difference and 95% confidence intervals (CIs) will be used. For enumeration data, risk ratio and 95% CIs will be calculated.

#### 2.5.5 Assessment of heterogeneity

The statistical heterogeneity will be examined by *I*^2^ test. When *I*^2^ ≤ 50%, heterogeneity is acceptable, and a fixed-effects model will be adopted, while when *I*^2^ > 50%, heterogeneity is obvious, and a random-effects model will be employed.

#### 2.5.6 Data synthesis

We will employ RevMan 5.3 (Cochrane Community, London, UK) software to synthesize and analyze the data, and to perform a meta-analysis if possible. If acceptable heterogeneity is examined among included trials, we will conduct a meta-analysis in accordance with the few variations in study and patient characteristics, and few differences in treatments, controls, and outcomes. If considerable heterogeneity is identified, we will carry out subgroup analysis and sensitivity analysis to find out any possible sources of obvious heterogeneity. If it is impossible to undertake a meta-analysis, we will report study results as a narrative summary.

#### 2.5.7 Publication bias

When the eligible studies are sufficient (over 10 RCTs), the reported bias will be visualized by funnel plot and Egger regression test.

#### 2.5.8 Subgroup analysis

We will observe the source of considerable heterogeneity by subgroup analysis based on variations in study and patient characteristics, study quality, different interventions, comparators, and outcomes.

#### 2.5.9 Sensitivity analysis

We will perform sensitivity analysis to test the robustness and satiability of conclusions by removing low quality trials, and trials with small sample size.

### 2.6 Quality of evidence

The quality of evidence for all outcomes will be assessed using the grading of recommendations assessment, development, and evaluation (GRADE)^[34]^ mainly considerations including: risk of bias, inaccuracy, inconsistency, indirectness, publication bias, and results of assessment will be graded 4 levels: very low, low, moderate, and high level.

## 3 Discussion

Spinal Cord injury is a disease with a high rate of clinical disability. Therefore, it is the focus of clinical research to find effective ways to relieve the degree of SCI and improve the movement and sensation after injury. Hyperbaric oxygen therapy for SCI has been increasingly reported in recent years, but there is still a lack of high-quality meta-analysis. This study is expected to provide more systematic and comprehensive evidence for the application of hyperbaric oxygen, and offer reference for the treatment of patients with SCI.

## Author contributions

**Conceptualization:** Tong Li, Yiran Wang, Qiang Ran, Xiaohong Fan.

**Data curation:** Tong Li, Yiran Wang.

**Formal analysis:** Tong Li, Yiran Wang, Xiaohong Fan.

**Funding acquisition:** Xiaohong Fan.

**Investigation:** Tong Li, Qianchun Li, Botao Chen.

**Methodology:** Qianchun Li, Xiaohong Fan.

**Supervision:** Yang Yu, Leiming Jiang, Xiaohong Fan.

**Validation:** Yang Yu, Leiming Jiang, Xiaohong Fan.

**Visualization:** Tong Li, Yiran Wang.

**Writing – original draft:** Tong Li, Yiran Wang, Chaoqun Feng, Qianchun Li.

**Writing – review & editing:** Qiang Ran, Botao Chen, Yang Yu, Leiming Jiang, Xiaohong Fan.
